# Effect of rearing system (free-range vs cage) on gut and muscle histomorphology and microbial loads of Italian White breed rabbits

**DOI:** 10.5713/ab.23.0203

**Published:** 2023-08-23

**Authors:** Caterina Losacco, Antonella Tinelli, Angela Dambrosio, Nicoletta C. Quaglia, Letizia Passantino, Michele Schiavitto, Giuseppe Passantino, Vito Laudadio, Nicola Zizzo, Vincenzo Tufarelli

**Affiliations:** 1 Department of Precision and Regenerative Medicine and Jonian Area, Section of Veterinary Science and Animal Production, University of Bari Aldo Moro, 70010 Valenzano, Bari, Italy; 2 Department of Veterinary Medicine, University of Study of Bari Aldo Moro, 70010 Valenzano, Bari, Italy; 3 Italian Rabbit Breeders Association (ANCI-AIA), 71030 Volturara Appula, Foggia, Italy

**Keywords:** Gut, Microbial Shedding, Morphology, Muscle, Rabbit, Rearing System

## Abstract

**Objective:**

The growing consumers’ interest on animal welfare has raised the request of products obtained by alternative rearing systems. The present study was conducted to assess the influence of housing system on gut and muscle morphology and on microbial load in rabbits reared under free-range (FR) and cage system (CS).

**Methods:**

A total of forty weaned (35 days of age) male Italian White breed rabbits were allotted according to the rearing system, and at 91 days of age were randomly selected and slaughtered for the morphological evaluation of tissue from duodenum and *longissimus lumborum*. Morphometric analysis of the villus height, villus width, crypt depth, villus height/crypt depth ratio, and villus surface was performed. The microbial loads on hind muscle was determined by total mesophilic aerobic count (TMAC), *Escherichia coli* and *Enterobacteriaceae*; whereas, total anaerobic bacteria count (TABC) and TMAC, *E. coli* and *Enterobacteriaceae* was determined on caecal content.

**Results:**

Rearing system did not interfere with the duodenum and muscle histomorphology in both rabbit groups. Similarly, microbial load of caecal content showed no significant differences on the TABC and TMAC. Conversely, significant difference was found for *E. coli* strains in caecal content, with the lower counts in FR compared to CS rabbits (p<0.01). Microbiological assay of muscle revealed significant lower TMAC in FR vs CS rabbits (p< 0.05). All rabbit meat samples were negative for *E. Coli* and *Enterobacteriaceae*.

**Conclusion:**

Free-range could be considered a possible alternative and sustainable rearing system in rabbits to preserve gut environment and muscle quality.

## INTRODUCTION

Recently, consumers interest on animal farming practices and their ethical issues have raised the request for products obtained by alternative rearing systems that assure high animal welfare standards and thus high product quality. In turn, animal welfare is now considered one of the most important factors defining the quality of meat and meat products [1,2]. As a result, an increasing number of EU regulations on welfare of farmed animals have been risen. In 2017, the European Parliament introduced a resolution on minimum standards for the protection of farmed rabbits, including an indication to ban the use of cages and to instead adopt free-range (FR) systems for growing rabbits [3]. After submission of the ‘End the Cage Age’ initiative in 2020, the European Commission published a legislative proposal to phase out, and ultimately forbid, the use of cage systems (CS) for farm animals, including rabbits [4]. Furthermore, the European Food Safety Authority (EFSA) Panel on Animal Health and Welfare produced a scientific opinion with an overview of the major risk factors related with different rearing systems and their consequences on the behavior and welfare of the rabbits, enunciating that “*It is likely to extremely likely (certainty 66–99%), that the welfare of growing rabbits is lower in conventional cages compared to the other housing systems*” [5]. Previous research has demonstrated that housing systems significantly influence the well-being, productive performances, and meat quality traits in rabbits [6–11]. Moreover, Fetiveau et al [12] suggested that the capacity of rabbits reared outdoor to express their specific behaviours and the low incidence of digestive disorders were indicative of enhanced animal welfare. In conventional rabbit farming, digestive disorders are the major reasons for welfare impairment for the rabbit starting from three weeks of age [5]. Further, the gut health is strongly related to the absorptive efficiency of available nutrients in the small intestine. Moreover, it is well known that the enteric layer plays an important barrier function against infectious diseases of the host [13]. The EFSA enunciated that the main hazards of gastroenteric disorders in rabbits related to the housing systems are mainly due to restricted space, high stocking density, floor type, lack of roughage, and stress [5].

Recent findings indicated that preservation of the gut microbiota equilibrium and digestive immunity seems to be effective to improve the ‘natural’ resistance to enteric diseases. Besides pathogens the intestinal microbial population imbalance (dysbiosis) is a critical factor in the development of digestive disorders [14]. Previous research showed that the housing environment may influence the gut microbiota structure [15]. Hubert et al [16] found that FR environments induced a higher gut microbiota diversity, thus enhancing the development and maintenance of the intestinal barrier and the mucosal immune system. The rearing conditions can also influence carcass and meat quality traits. D’Agata et al [1] referred that the increasing of physical activity in rabbits reared in outdoor systems positively influences growth performance, carcass quality and meat quality traits. Increased movement can affect carcass traits and meat quality, through differences in muscle development and fat deposition [10, 17,18].

Therefore, the aim of this study was to investigate how alternative rabbit farming systems (FR vs CS) may influence gut homeostasis and morphology, and muscle structure development. In addition, microbiological analysis was also conducted on rabbit caecal content and to verify the possible interaction between housing condition and gut health, and to assess the hygienic meat status of rabbits.

## MATERIALS AND METHODS

### Animals and management

Rabbits in the present study were cared and handled in compliance with the EU legislation on animal welfare regulations (Directive 2010/63/EU, which updates and replaces the 1986 Directive 86/609/EEC) and following the research policies of the DiMePRE-J of the University of Bari Aldo Moro, Italy (Approval code: DiMePRE-J/07/2022). The research was conducted in an experimental rabbitry located in the province of Bari, Apulia region (Italy). Forty male Italian White (*Bianca Italiana*) breed rabbits, obtained from the Central Breeding Farm of the Italian Rabbit Breeders Association (ANCI-AIA, Volturara Appula, Foggia, Italy) and aged 35 days (body weight 1,045±10.1 g, mean± standard error of the mean), were randomly assigned to two groups of 20 animals according to the rearing system: FR and CS as in our previous study [11]. Briefly, within each group, rabbits were divided into five replicates having four rabbits/replicate, for a total of 20 rabbits per group. The trial lasted up to 91 days of age. Rabbits in CS were housed individually under standard conditions between 15°C–23°C, controlled by heating and forced ventilation systems, in wire cages measuring 360× 450×310 mm and at a height of 90 cm from the concrete floor. The rabbits reared in the FR system had a whole-day access to the range and were shepherded to the same house. Under FR conditions, the available space for rabbits was 0.25 m^2^/head, so that each area available for replicate having four rabbits was 1 m^2^; also, the area was composed by a 3 m high metal fence protected by a shade net to deny access to possible predators. Four points of feeding were supplied in each area under a plastic cover. In the FR area there was no grass but only shelters and trees, so that no supplemental feed was available to rabbits under FR system. Rabbits of both groups were fed *ad libitum* and water was freely available from nipple drinkers. The ingredients composition and chemical analysis of diet is shown in Table 1. No medication was included in the feed or in the drinking water and rabbits’ health status was checked through individual observations. At the end of the fattening period (91 days of age), ten rabbits per group were randomly selected in the afternoon for slaughter. On the next morning, the selected rabbits were transferred in small groups to the slaughter facility near the experimental building to determine carcass traits. The rabbits were then weighed, electrically stunned, and slaughtered within 2 h. The slaughtering and sampling procedures followed the World Rabbit Science Association (WRSA) recommendations as described by Blasco and Ouhayoun [19].

### Morphological measurement of duodenum

After slaughter, a 3-cm segment of duodenum from ten subjects in each group were collected and fixed immediately using neutralized 10% (v/v) formalin and embedded in paraffin. A 5 μm thick sections were cut from paraffin blocks, mounted on slides, and stained with hematoxylin-eosin (H-E) for morphometric examination and duodenal mucosa morphology. For each rabbit, 5 images were captured from each slide, and minimum of 5 villi were measured for length and width, and crypt depth. The villus height was measured from the villus tip to villus–crypt junction level for 5 villi per section, the width was measured at the half height point. Calculation using villous height and width at half height gave the villus surface area. Crypt depth was measured from the villus–crypt junction to the lower limit of the crypt and it was estimated for 5 corresponding crypts per section. The villus to crypt ratio was also calculated for each segment. The mucosa and muscular layer thickness were also measured. The morphometric measurements were taken with a camera HD (DS-Fi2 high-definition color camera; Nikon Corporation, Tokyo, Japan) connected to a light microscope (Nikon Eclipse Ni-U; Nikon Corporation, Tokyo, Japan) and measured by an imaging system software (NIS Elements BR; Nikon Corporation, Japan).

### Muscle morphology evaluation

Samples from the *longissimus lumborum* muscle were dissected intact from the origin insertion then were cut into segments of almost 1 cm^2^ and were fixed in 10% neutral buffered-formalin. Fixed samples were placed in cassettes and soaked in formalin and alcohol of different concentrations using Histokinette device 2000 for the fixation and dehydration of muscle tissues. Then dehydrated tissues were routinely embedded in paraffin at 75°C using a paraffin dispenser while still in cassettes. After wax infiltration the tissue samples were orientated in the cassettes in the same direction. Sections 5 to 7 μm thick were cut from paraffin blocks using a rotary manual microtome (RM2235; Leica, Milan, Italy), mounted on slides and stored at room temperature. Slides were dewaxed in xylene, hydrated using graded ethanol, and stained for routine histological evaluation by H-E staining (Merck, Darmstadt, Germany), Azan Mallory and Mallory blend (Merck, Germany), for morphological observations by 25×, 250×, and 400× magnification, using an image analysis system (X-Series, Alexasoft).

### Meat and caecal microbiological evaluations

From the same rabbit carcasses, samples of hind muscle were taken. The following microbiological analysis were performed: total mesophilic aerobic count (TMAC), *Enterobacteriaceae* count and *Escherichia coli* (*E. coli*) β-glucoronidase-positive count. A 30 g of rabbit meat was added to 270 mL of buffered pepton water (BPW) (Liofilchem, Teramo, Italy), homogenized in stomacher (Lab-Blender 400; PBI, Milan, Italy) for 2 min, and decimally diluted in BPW for microbial enumeration. For TMAC each dilution was pour plated on plate count agar (PCA) (Liofilchem, Italy) and incubated at 30°C±1°C for 72±3 h. For *Enterobacteriaceae* count, 1 mL of each decimal dilution was pour plated on Violet Red Bile Glucose Agar (Conda, Italy). For *E. coli* β-glucoronidase-positive count, 1 mL of each decimal dilution was pour plated on Tryptone Bile X-Glucoronide Agar (Biokar Diagnostic, Beauvais, France) and incubated at 44°C±1°C for 18 to 24 h.

About 100 g of caecal content samples were collected, and 10 g were added to 90 mL of BPW (Liofilchem, Italy) homogenized in stomacher (Lab-Blender 400; PBI, Italy) for 2 min, and decimally diluted in BPW for microbial enumeration. For total anaerobic bacteria count (TABC) 0.1 mL of each dilution was spread in BD Schaedler Agar with 5% sheep blood supplemented with Kanamycin and Vancomicin (Liofilchem, Italy) and incubated in at 35°C to 37°C, under anaerobic condition, for at least 48 h and up to 7 days. For TMAC, 1 mL of each dilution was pour plated on PCA (Liofilchem, Italy) and incubated at 30°C±1°C for 72±3 h. For *E. coli* count, 0.1 mL of each dilution was spread on MacConkey Agar (Liofilchem, Italy) and incubated at 35°C ±1°C for 24 to 48 h.

### Statistical analysis

Data were analysed by one-way analysis of variance using the general linear model procedure of SAS Institute Inc. Software. Each replicate within treatment was considered as experimental unit. For microbiological evaluations, data were expressed as log colony-forming unit (CFU)/g to detect possible significant differences on microbiological loads. Data are presented as least-squares means and the difference among means was tested by Tukey’s test. A level of p<0.05 was used as the criterion for statistical significance.

## RESULTS

### Intestinal morphology

**Figure 1:**
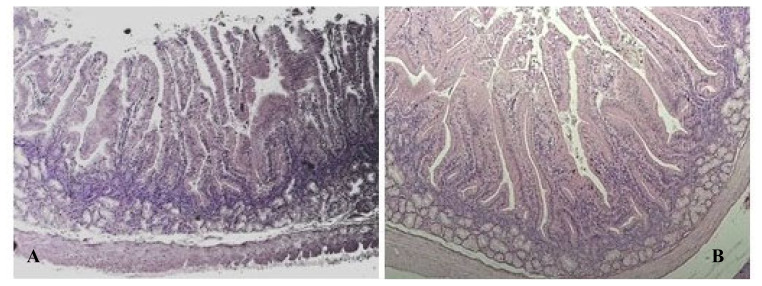
Histological sections of duodenum collected from free-range (A) and caged (B) rabbits (Hematoxylin and eosin; 25×). A normal architecture of the intestinal layers was observed in both groups. Microscopic observation revealed normal columnar cells and scattered goblet cells secreting a protective layer that lines the surface of the epithelium being attached on a regular mucosal muscle layer. Morphometric evaluation discovered properly development of the villi and the gland components in both groups.

Morphological examination of the duodenum segments of the studied groups showed conserved, typical, and shapely structure and no significant difference was found between the histological features of rabbits reared in FR vs in CS. Figure 1 shows a representative histological cross section of duodenum stained with H&E. As seen in Figure 1 the luminal surface of the small intestine was covered by villi that displayed a regular structure, composed of columnar absorptive epithelial cells, enterocytes, and interspersed goblet cells that line the mucosa wall. The lamina muscularis, that separating the tunica mucosa from the submucosa, and the submucosa showed overlapping thickness and the absence of histopathological features in both the groups. The microscopic observation of tissue samples also revealed an appropriate development of the intestinal gland component that extends from the muscularis mucosa through the thickness of the lamina propria and opens into the intestinal lumen at the villi base. The Brunner glands were massively present in the cranial part of the duodenum compared to the other parts [20]. Also, morphometric analysis of the villus height, villus width, crypt depth, villus height/crypt depth ratio, and villus surface area indicated that there were no significant differences (p>0.05) between FR and CS rabbits (Table 2).

### Muscle morphology

**Figure 2:**
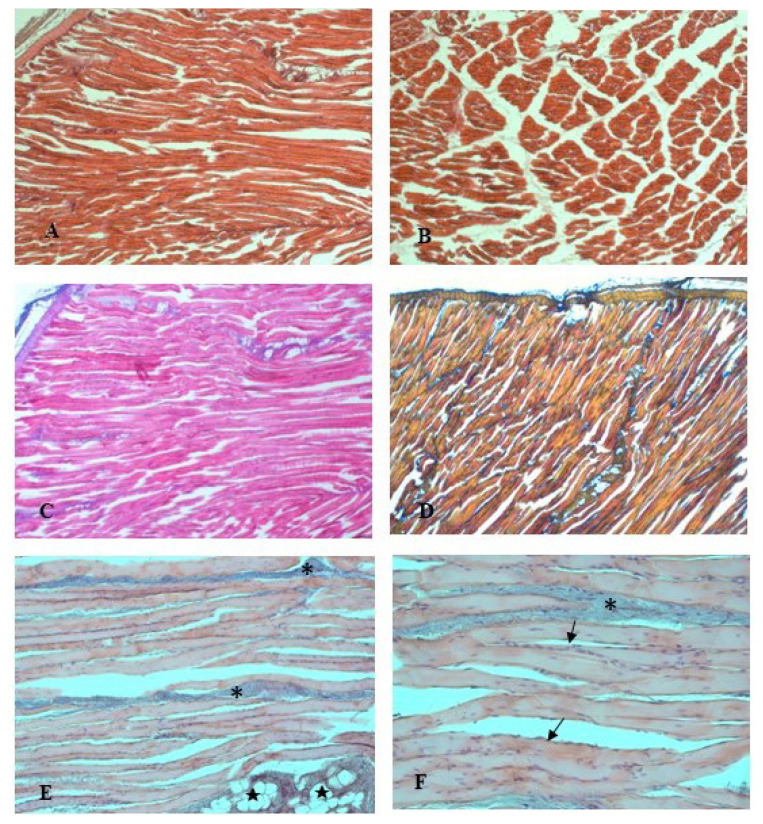
Photomicrographs of rabbits *longissimus lumborum* muscle at longitudinal (A) and cross (B) sections (Hematoxylin-Eosin, 25×). The connective tissue is highlighted by the trichromic stains, Azan Mallory (C, 25×) and Mallory blend (D, 25×), in shades of blue. High magnification light micrographs, stained by Azan Mallory, show portions of fibres separated by perimysium (*): fat lobules (★) and blood vessels are visible (E, 200×); flattened nuclei (arrows) lie just beneath the sarcolemma of the fibres (F, 400×).

The examination of the housing system effects on the histological traits of rabbits’ *longissimus lumborum* muscle found no difference between the two groups. The micro-analysis showed normal muscle fibers with their connective tissue elements, and no structural changes were observed. The fiber type distribution was conserved. Morphological observations of the connective tissue, endomysium and perimysium, with the thrichromic staining techniques (Azan Mallory and Mallory blend, Figure 2C–2D) showed a thin delicate layer of reticular fibers that surrounds the muscle fibers. In the muscle from FR rabbits, the fibers showed uniform width and regular oval-shaped nuclei, typically located at the periphery of the cells (Figure 2E–2F). Scarce fat lobules and blood vessels are also visible in Figure 2E. In the longitudinal sections, the muscle fibers displayed a normal arrangement in linear myofibrillar structure and had a stripy appearance, because of the repeating structure of the muscle (Figure 2A). In the cross sections, myofibers presented homogeneous diameter and reveal their characteristic polygonal shape.

### Microbial analysis

**Figure 3:**
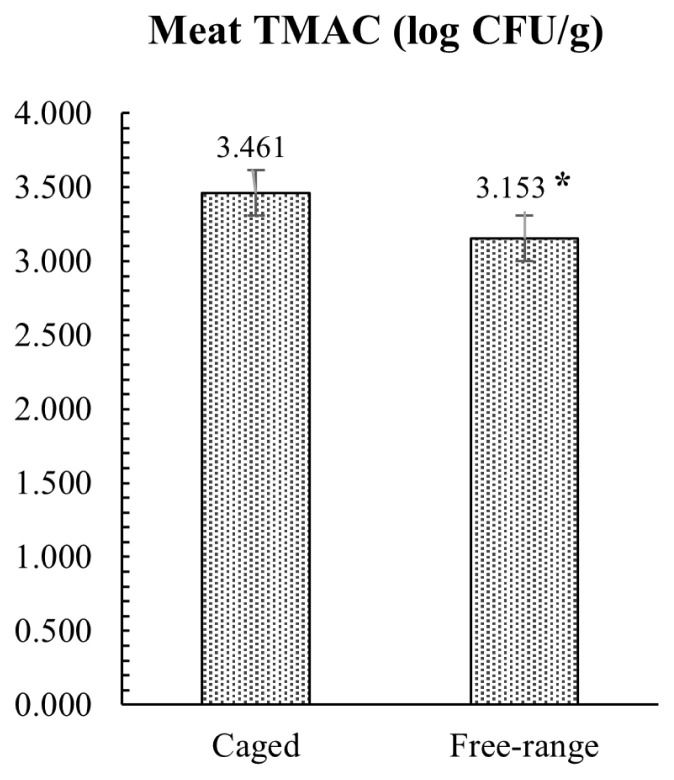
Effect of rearing systems on total mesophilic aerobic count (TMAC) in rabbit meat muscles.* p<0.05.

**Figure 4:**
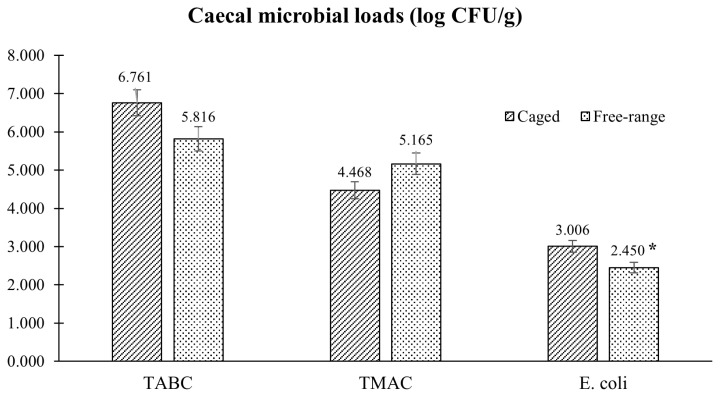
Effect of rearing system on total anaerobic bacteria count (TABC), total mesophilic aerobic count (TMAC) and *Escherichia coli* in rabbit caecal content. * p<0.05.

The results of the microbiological investigation on hind meat muscle samples are presented in Figure 3. Microbial analysis revealed that FR rabbits had lower TMAC compared to the group reared in conventional cage, with a mean value of 3.15 and 3.46 log CFU/g, respectively (p = 0.012). All rabbit meat samples analyzed were negative for the enumeration of *E. coli* β-glucuronidase-positive and *Enterobacteriaceae*. The caecal microbial loads conducted on TAMC, TABC, and *Enterobacteriaceae* showed similar results with no statistical differences between the two tested groups, as showed in Figure 4. Conversely, *E. coli* load was significantly lower (p = 0.002) in FR rabbits.

## DISCUSSION

Small intestinal morphology is one of the main criteria used for the evaluation of the intestinal physiology [21]. Moreover, the morphometric assay, villus height, crypt depth, villus height/crypt depth (V/C) ratio, and villus surface area, are usually employed for the evaluation of the digestive and absorptive capacity of the intestine. Our results indicated that the FR housing did not have any effects on the intestinal histomorphological features of rabbits, showing overlapping aspects in both groups analyzed and suggesting that the outdoor rearing provides an adequate development of the enteric structural components. Similarly, no significant differences were found on the TABC and TMAC caecal content in the studied groups. Otherwise, microbial loads showed significant difference for the *E. coli* count, with the lower content in the FR rabbits (2.45 and 3.01 log CFU/g in FR vs CS rabbits, respectively; p<0.05). The most common disorder in rabbit production is the occurrence of enteritis. *E. coli* and *Clostridium* spp. are two potential pathogenic bacteria frequently present in diarrheic rabbits and can lead to mortalities after weaning in excess of 20% [22,23]. Broadly, the term “gut health” describes the interaction between the intestinal wall barrier, the microbiota, and the immune components, which permit organisms to cope with internal and external stressors [24,25]. It is well known that the intestinal wall represents a natural barrier against pathogens and toxic substances present in the intestinal lumen. Also, the intestinal microbiota plays an important role in metabolic, nutritional, physiological and immunological processes [26]. Previous research has shown that the housing environment may influence the gut microbiota structure in livestock species. Schreuder et al [15] referred that in laying hens a cage-free system generated higher gut microbiota diversity compared to caged layers, and that the diet had a relatively lower effect on changing the gut microbiota, suggesting that the outdoor access and contact with soil and natural vegetation are likely important in raising gut microbiota diversity [27]. As considered by Round and Mazmanian [28] and Lee et al [29], greater microbial gut variability translates into improved immune and metabolic performances. Our hypothesis was that the lower *E. coli* caecal count found in rabbits reared under FR conditions could be related to an increase of gut microbiota diversity as well as to the improvement of gut health and well-being that enhanced the natural function of the intestinal barrier against pathogens.

In order to produce high-quality meat, it is necessary to understand the characteristics of meat quality traits and factors to control them [30]. The housing system is one of the factors, which moderately affect rabbit carcass and meat quality [31]. It should be enounced that meat comprises numerous tissues such as adipose, epithelial, connective and nervous tissues, even as the major component is muscle, thus the study of the muscles’ microscopic structures may provide useful information about the meat quality traits. Myofiber structure, diameter and organization, and collagen structure, thickness and distribution, have shown an important influence on the meat quality traits, indeed numerous studies have shown the relationship between meat quality attribute and fiber characteristic [30]. Total number of fibers (TNF) and cross-sectional area of fibers (CSAF) are the primary morphology traits that influence the development of muscle mass as well as the meat quality [29]. Also, contractile and metabolic assets of muscle are linked to fiber type composition (FTC) in muscle [32,33]. Moreover, the muscle fiber characteristics are significant for growth performance, for instance, Lee et al [34] shown that the TNF and CSAF are significantly correlated with growth rate and carcass productivity of examined pigs. The meat sensory properties are also influenced by various structural properties of the muscle tissue like intramuscular fat (IMF) content and spatial organization, collagen content and spatial organization, myofibers spatial organization, type, size, shape and density [35]. In particular, the meat fibers and the spatial organization of the conjunctive network of fat, which defines the “meat grain”, are one of the meat structure traits firmly related to meat tenderness. Moreover, meat texture and firmness are also influenced by the size of muscle fiber, the amount of connective tissue, and the quantity of subcutaneous and IMF [30]. Among others, myofibrillar structure is highly influenced by the animal rearing conditions [35]. Greenwood et al [36] found that single- or multiple-reared lambs present significant differences in myofiber types, whereas Gondret et al [18] reported changes in myofiber types according to indoor or outdoor rearing systems in rabbits. Recent knowledge underling the importance of assess the transition mechanisms that influence the FTC. Furthermore, it has now long been proven that physical activity positively affects this parameter [30]. The more space available and the greater freedom of movement in outdoor housing conditions increased the physical activity of rabbits. According to Lefaucheur and Vigneron [37], the FTC can be changed by physical exercise, depending on the type and duration of the activity. In addition, Ouhayoun [38] referred that the increased movement affects muscle fiber type and size, which can increase the proportion of so-called “red” to “white” muscle fibers, which differ in their mitochondria or myoglobin content, and can affect the colour of the meat. Indeed, the exercise raises the oxidative capacity of the muscle, which increases the proportion of oxidative myofibers and the myoglobin content, thus influencing the meat redness [18]. Accordingly, Krunt et al [10] observed also increases in redness of the *Quadriceps femoris* muscle in pen-housed rabbits. The increase in meat redness can be explained by the fact that as animal movement increases the number of mitochondria in αW fibers, converting their predominant glycolytic energy metabolism into oxidative energy metabolism and then, part of the αW fibers turn into αR fibers, richer in myoglobin [39]. In contrast, reduced movements increase the muscle glycogen storage used for the anaerobic energy metabolism [38]. The greater development of the hind part of the carcass of rabbits with more opportunities for physical activity has also been reported by other studies investigated housing systems which allow for different degrees of physical activity [6,40]. Gondret et al [18] proved that subjecting rabbits to jumping exercises for 5 weeks significantly increased the development of the hind parts compared to rabbits that were not exercised. D’Agata et al [1] referred that increasing physical exercise in FR housing raised the development of rabbit hind legs. It was theorized that the augment in physical activities could impacts the sizes of the muscles tissue, thereby affecting features such as yield, colour, and shear force [10]. Thus, the alternative housing system, complying with the conditions of animal welfare, well fit the increasing consumers demand for home-made products and high-quality animal products [2]. Our results showed that the FR system did not influence the muscle structure, and the histological assay of the spatial organization and the composition of muscle samples from FR rabbits satisfied the major parameters related with the meat quality traits.

Furthermore, as demonstrated by the low microbial counts, the sample of muscles tested in this study showed an optimal microbiological quality at slaughter, with lower TMAC in FR vs CS rabbits, and the absence of the *Enterobacteriaceae* and *E. coli* strains in all the samples analyzed. The initial microbial load of meat is influenced by the physiological status of the animal and by the hygienic state during slaughter, and production processes [41]. Moreover, Pereira and Malfeito-Ferreira [42] have highlighted the importance of a low microbial count on rabbit meat shelf-life, assuming that also growth parameters are influenced by the initial contamination [43]. In addition, rabbit meat is more prone to lipid oxidation than other meats, and it can easily permit the growth of pathogenic and spoilage microorganisms [42, 44]. Tufarelli et al [11] found that muscles from FR rabbits showed an improvement in the oxidative stability in respect to the group reared in conventional cage. In particular, the meat from FR rabbits had a lower thiobarbituric acid-reactive substances level compared to CS rabbits, suggesting that the housing system may fortify the meat oxidative stability. It’s well known that environmental stress may influence the oxidative processes in the body, disrupting the balances between oxidative-antioxidative reactions and leading to an increase of production of reactive oxygen species that progress the detrimental oxidative changes in organic tissue. In turn, the meat oxidative stability influences the shelf life and the microbiological quality of the muscles. In the present study, the lower TMAC and the absence of *Enterobacteriaceae* and *E. coli* strains found in rabbits reared in FR system suggested that this rearing system allows an improvement of the hygienic conditions and rheological characteristics of meat that could be also related to a reduction of stress as well as an increase of rabbit well-being promoted by the alternative farming system.

## CONCLUSION

Over the years the rabbit rearing systems have gained the attention of scientific researches in order to improve well-being and also to obtain high-quality products. Rearing systems combined with high standards of animal welfare resulted in a better quality and safety of the final products. Access to FR can improve welfare and permit animal to show innate behaviors. The present study demonstrated that rabbits reared under FR condition had similar results compared to CS animals, suggested that the alternative system did not negatively interfere with the physiologic gut and muscle architecture and function. Moreover, microbiological loads revealed an improvement of gut health and meat hygienic status in FR rabbits. However, further studies are needed to deeply understand how the housing system may influence animal health and production.

## Figures and Tables

**Table 1:** Ingredients and chemical composition of the diet fed to rabbits

Items	
Ingredients (g/kg diet)	
Dehydrated alfalfa meal	285
Dehydrated beet pulp	285
Corn	200
Soybean meal, 48% crude protein	100
Wheat middlings	84.5
Cane molasses	20
Vitamin-mineral premix^1)^	50
Monocalcium phosphate	50
Sodium chloride	40
Calcium propionate	25
L-lysine	25
DL-methionine	25
Yeast	10
Magnesium oxide	10
Magnesium carbonate	10
Chemical composition (g/kg as-fed)	
Dry matter	891
Crude protein	154
Ether extract	24
Crude fibre	141
Neutral detergent fibre	268
Acid detergent fibre	167
Lignin	39
Ash	69
Digestible energy (MJ/kg)^2)^	10.61

1) Provided per kg of diet: vitamin A 12,500 IU; vitamin D_3_ 1,500 IU; vitamin E 30 mg; vitamin B_1_ 1.5 mg; vitamin B_2_ 5 mg; vitamin B_6_ 2 mg; vitamin B_12_ 0.02 mg; vitamin PP 20 mg; vitamin K_3_ 2.5 mg; folic acid 0.75 mg; pantothenic acid 10 mg; D-biotin 0.1 mg; choline chloride 300 mg; MnSO_4_ 150 mg; FeSO_4_ 5 mg; ZnSO_3_ 75 mg; CuSO_4_ 5 mg; KI 1 mg; CoSO_4_ 0.2 mg; Na_2_SeO_3_ 0.1 mg.

2) Calculated as: 12.912 – (0.0236 × crude fiber) + (0.010 × crude protein) + (0.020 × ether extract).

**Table 2:** Effect of rearing system on duodenal histomorphometry of rabbits

Item	Conventional cage	Free-range	Pooled SEM	p-value
Villus height (μm)	967	1,047	38.4	0.062
Villus width (μm)	72.8	81.9	3.13	0.059
Crypt depth (μm)	91.3	129.5	4.05	0.067
Mucosa thickness (μm)	159.8	190.3	4.89	0.055
Muscular thickness (μm)	17.98	18.71	1.952	0.081
Villus height/crypt depth	10.59	8.08	1.041	0.058
Villus surface area (mm^2^)	0.101	0.116	0.070	0.063

Each value represents the mean of ten rabbits per group.

SEM, standard error of the mean.
